# Model of Illusions and Virtual Reality

**DOI:** 10.3389/fpsyg.2017.01125

**Published:** 2017-06-30

**Authors:** Mar Gonzalez-Franco, Jaron Lanier

**Affiliations:** Microsoft ResearchRedmond, WA, United States

**Keywords:** virtual reality, embodiment, perception, cognition, avatars

## Abstract

In Virtual Reality (VR) it is possible to induce illusions in which users report and behave as if they have entered into altered situations and identities. The effect can be robust enough for participants to respond “realistically,” meaning behaviors are altered as if subjects had been exposed to the scenarios in reality. The circumstances in which such VR illusions take place were first introduced in the 80's. Since then, rigorous empirical evidence has explored a wide set of illusory experiences in VR. Here, we compile this research and propose a neuroscientific model explaining the underlying perceptual and cognitive mechanisms that enable illusions in VR. Furthermore, we describe the minimum instrumentation requirements to support illusory experiences in VR, and discuss the importance and shortcomings of the generic model.

## Introduction

As it is the case with many technologies, the beginnings of VR are closely linked to industry and startups. It is the manufacturing of devices that popularizes the technologies, making it available for others. In that regard, despite the initial concept of VR was formulated in the 1960s by Dr. Ivan Sutherland, it wasn't until later that the first devices became available. One of the authors (Lanier) lead the team that implemented the first experiences with avatars and social virtual reality (VR) (Lanier et al., 1988; Blanchard et al., 1990). This work occurred in the context of a 1980s technology startup (VPL Research), and while results were reported in the popular press (Lanier, 2001) and anecdotally, the context was not one in which rigorous experiments were undertaken, nor was research peer reviewed (Lanier, 1990). VPL Research provided initial VR instrumentation for many laboratories and pioneered a school of thought that described some of the many possibilities of avatars and VR for social and somatic interactions (Blanchard et al., 1990). Meanwhile, in the intervening decades, the original hypotheses have been refined and empirically formalized by the scientific community (Blascovich et al., 2002; Tarr and Warren, 2002; Sanchez-Vives and Slater, 2005; Yee et al., 2009; Bohil et al., 2011; Fox et al., 2012). Reflecting on this body of research, we can gain a general understanding of illusions that take place in VR. In this paper, we not only review a broad range of VR illusions, but also propose a comprehensive neuro-perceptual model to describe them.

Our proposal integrates and explains a wide variety of VR illusions that have been formally investigated through a combination of three classes of processes borrowed from established neuroscience models: bottom-up multisensory processing (Calvert et al., 2004; Blanke, 2012), sensorimotor self-awareness frameworks (Gallagher, 2000), and top-down prediction manipulations (Haggard et al., 2002). Using this model, we can understand the perceptual and cognitive mechanisms that trigger the great majority of illusions in the literature of VR.

## Illusions enabled by virtual reality

While VR instrumentation varies, it always includes sensors to track and measure a set of the person's body motions, such as the motion of the head, and often a great deal more about the person's physiological state, including pose, force, metabolic, or interoceptive factors, and so on, as well as an equally variable set of actuation and display devices. VR could, at a hypothetical extreme, measure anything in the human body, and present a stimulus for any sensory modality of the human body. VR sensors are typically paired with VR displays or actuators. For instance, if a display device addresses a sensory modality located in the human head, such as the eyes or ears, then head tracking becomes relevant.

When these sensor-coupled stimuli match the brain's expectations of what the next moment will bring, then the brain will tend to treat the simulated reality as real, which in turn will engage additional neural mechanisms to further the veracity of the illusion. Indeed, the everyday perception of physical reality relies on a low-level, continuous calibration of raw data from biological sensors, which might be thought of as mild, continuous hallucinations, or imperfect implicit neural hypotheses of what to expect from the real world. These are constantly corrected based on new input to enhance the perceived veracity of a virtual world (Lecuyer, 2017).

The popular literature of the 1980s described a “conversion moment”—that took place a second or two after a user donned a headset—when a VR user stopped responding to the physical environment, and started to experience the virtual world as effectively real. It is possible that this sense of a slightly delayed conversion moment was more noticeable with the cruder equipment of that period. It continues to be the case that there is a transition during which a user shifts awareness and behavioral responses to the virtual world instead of the physical. This is not unexpected since other types of multisensorial illusions that do not require VR, such as the Rubber Hand Illusion, also take time to elicit (Botvinick and Cohen, 1998).

The effect has been compared—in popular culture—to a hallucinogenic drug experience. However, illusory states in VR don't *directly* alter higher cognitive functions, as happens when chemically stimulating the brain with hallucinogenic drugs. Nonetheless, VR users can feel that they have been transported to a new location (place illusion), that the events happening are real (plausibility illusion) (Sanchez-Vives and Slater, 2005), and even that their bodies have been substituted by an avatar (embodiment illusion) (Spanlang et al., 2014).

Indeed, it is because VR illusions are driven by the neurological mechanisms of everyday perception of the body in the world that participants often exhibit realistic responses to VR (Slater, 2009). For instance, participants prefer to take a longer path on (simulated) solid ground rather than walking over the famous illusion of a virtual pit (Meehan et al., 2002). The responses to the virtual pit are so realistic that increases in heart and respiratory rate are registered when approaching the void.

Human cognition is highly attuned to other people in the physical environment and this remains so in virtual environments. The study of avatars in VR is therefore central to the understanding of cognition and behavior in VR.

Participants not only respond realistically to the environment, but also behave genuinely when interacting with avatars. Despite the challenges of the uncanny valley, avatars are processed in the brain like people, and humans are able to recognize differential familiarity levels on avatar faces (Bailenson et al., 2006; Gonzalez-Franco et al., 2016). Hence, social norms, such as interpersonal distance, are kept when interacting with avatars (Bailenson et al., 2003; Sanz et al., 2015). In the same way, more complex social behaviors are also reproduced inside VR: shy males show higher anxiety when interacting with a virtual female than confident males (Pan et al., 2012), or self-similar avatars (Aymerich-Franch et al., 2014). And, people immersed as bystanders during violent incidents in VR are likely to intervene following realistic behavioral patterns (Rovira et al., 2009).

The full-body illusion is a phenomenon unique to VR (Lanier et al., 1988). It takes place when participants feel they inhabit a virtual body (Heydrich et al., 2013). This experience can be induced by presenting a virtual body co-located to the participant's body (Maselli, 2015).

The effect can be enhanced by presenting a VR mirror to the participants in which they can see their virtual body moving as they move from a first person perspective (Gonzalez-Franco et al., 2010), but also through passive visuo-tactile multisensory stimulation (Kokkinara and Slater, 2014). A virtual body (AKA an avatar body) enhances the exploration and interaction capabilities of VR in an ergo-centered fashion. Participants not only gain a visual representation so that they can socialize, but also have access to a wider set of methods of interacting with the simulated world.

Interestingly, changing the design of a virtual body can elicit behavioral changes (Bailenson and Segovia, 2010; Fox et al., 2012). For example, participants altered the way they play music depending on the embodied avatar, being less musical when the avatar was dressed as a business man (Kilteni et al., 2013). Test subjects also modified their behavior during psychological treatment when embodying an avatar representing Sigmund Freud (Osimo et al., 2015).

The link between avatar design and behavior is probably related to pre-conceived stereotypes and mimicry effects. Humans easily interiorize stereotypes associated with their life experiences and what they learn from the environment, producing unconscious biases that influence behavior when exposed to new situations (Bourgeois and Hess, 2008). Those mechanisms mix with non-conscious mimicry during social interactions. Mimicry is well-known to be elicited as an automatic behavior in response to social exclusion and to reduce outgroup effects (Lakin et al., 2008). Indeed, the human desire to fit in and be liked can not only alter personalities, but might be so profound as to alter one's own physiological interoceptive function to reflect an interlocutor during conversation (Durlik and Tsakiris, 2015).

The mimicry effect in VR and its relationship with preconceived stereotypes is well illustrated in the research of Prof. J. Bailenson that investigates how participants assimilate nonverbal gestures and behaviors through imitation in immersive VR (Bailenson and Yee, 2005; Fox et al., 2009). Sometimes this effect can produce positive outcomes, such as increased empathy (Rosenberg et al., 2013), but in other occasions it might lead to self-objectification in a sexualized context (Fox et al., 2013). Through avatar design and virtual scene changes, VR enables the study of non-conscious mimicry and personality altering effects with a reduction of unknown environmental variables. For instance, an improvement of negotiation skills has been observed when a subject is embodied in a taller avatar (Yee and Bailenson, 2007). More mature financial decisions were evoked when subjects inhabited avatars that approximated aged versions of themselves (Hershfield et al., 2011).

Aside from behavioral changes, subjects can also accept substantial structural transformations to their virtual bodies, even temporarily altering self-body perception (Normand et al., 2011).

This effect was first observed in the 1980s, and was dubbed Homuncular Flexibility (Lanier, 2006). Formal study of Homuncular Flexibility has confirmed the earlier, informal observations (Won et al., 2015a). An example of this effect is that participants embodied in differently shaped avatars can overestimate their own body size (Normand et al., 2011; Piryankova et al., 2014).

A remarkable result is that subjects can be made to naturally accept supernumerary limbs (Won et al., 2015b). For instance, subjects can control tails on their avatars (Steptoe et al., 2013). Furthermore, being inside an avatar with a full-body ownership illusion, in which one feels that the virtual body is her body, might elicit self-attribution mechanisms. Those mechanisms enable for an action of the avatar to be incepted in the brain as being originally intended by the participant, producing an illusory sense of agency. Test subjects can self-attribute small alterations in their motor trajectories (Azmandian et al., 2016) and even in the speech of an avatar (Banakou and Slater, 2014). However, sufficiently radical alterations of avatar actions cause semantic violations and are rejected by testers (Padrao et al., 2016). In this sense, VR can contribute to the better understanding of the brain's plasticity, and help explore how the brain and the body integrate by presenting scenarios beyond what would be physically feasible.

## Minimum instrumentation requirements to support illusory mechanisms in virtual reality

Illusory experiences are not only a consequence of using VR, but the very foundation of its operation. In VR, the participant is not merely an observer, but is the center of the system, both screen and viewer. In order to enable this self-centered experience, plausible sensory stimulation must persuade the brain that realism has not been lost when natural information derived from the physical environment is replaced by computer generated information. This process of successful substitution enables VR experiences to “feel real” (Brooks, 1999; Guadagno et al., 2007; Slater, 2009).

Complex VR systems incorporate congruent stimulation of multiple modalities such as vision, audition, and tactile/proprioception (the latter typically when participants are represented by a virtual body). Evidence shows that VR can successfully stimulate coordinated human perceptual modalities so that brain mechanisms which collect and process afferent sensory input will interpret the data coherently (Kilteni et al., 2015).

A useful definition of VR, which distinguishes VR from other complex media technologies, is that VR tends to avoid semantic violations as the brain and body interact in synch with the simulated environment. As an example, we can consider “spherical videos” which are commonly available on headsets that make use of smartphones which include sensors for rotation, but not for translation.

Despite the utility of stimulating multiple sensory modalities to engage the integration that enhances a fully ergo-centered experience, one particular sense has remained key to VR: vision. Visual dominance is a human characteristic (Posner et al., 1976); therefore it is not surprising that visual input is exceptionally important to VR.

In that sense, stereoscopic photography (dating to the 1840s) can be considered a precursor to VR. A pair of photographic prints aligned for typical human interocular distance, mounted on a stereoscope with a sufficient Field of View (FoV) and accommodation can create a minimal, self-experiential illusion capable of briefly transporting users to an alternate reality. Static stereoscopic photography has since evolved into spherical videos.

Illusory states can be convincing in spherical video technologies, but only provided that users do not try to interact with the environment. These relatively passive experiences (with no translational motion, very limited interactivity, and without body representation) can generate realistic brain responses; e.g., motor cortex activation is found even in static setups when a virtual object attacks a static participant in VR (González-Franco et al., 2014).

However, since there is no underlying dynamic simulation that can respond to variations in user behavior, this type of illusion breaks the moment users try to explore or interact with the virtual environment, constraining the veracity of the self-centered experience, and engendering a “body semantic violation” (Padrao et al., 2016). Therefore, the minimal instrumentation required to produce the illusion of entering VR without semantic violations (i.e., breaks on the illusion) must combine a continuously updated (head tracked, at a minimum) display with congruent sensorimotor contingencies (Spanlang et al., 2014).

This principle can be generalized. We can evaluate whether a given media technology instrumentation can be understood as VR by how well it avoids semantic violations. While there might never be an instrumentation for VR that completely avoids semantic violations, there are many designs for VR hardware in which a user will typically not encounter a semantic violation for extended periods of time. The authors acknowledge that this is a subtle issue that might be understood somewhat differently in the future due to cultural change or shifting philosophical interpretations, but nonetheless, a practical difference between systems that display semantic violations almost immediately and those that largely avoid them as been demonstrated.

## Toward a cognitive model: which brain activities facilitate virtual reality illusions?

The underlying brain mechanisms that enable users to “believe” that a computer-generated world is effectively real can be modeled through a combination of at a minimum three classes of processes: bottom-up multisensory processing (Calvert et al., 2004; Blanke, 2012), sensorimotor self-awareness frameworks (Gallagher, 2000), and top-down prediction manipulations (Haggard et al., 2002). We first consider bottom-up multisensory processing.

Bottom-up sensory processing is understood as an aggregated probabilistic cognitive strategy. The brain combines bodily signals subject to a degree of noisy variation in weighting and other parameters, and adapts those parameters continuously based on feedback. It can be framed as a natural analog to artificial sensor fusion. Signals arrive from different modalities with different temporal and spatial resolutions, different degrees of freedom, and presumably differences in coding, but the brain is able to integrate them effectively.

Bottom-up sensory processing implicitly infers the most effective ways to respond to the external world from moment to moment, but is also a key aspect of self-body consciousness (Blanke, 2012). Another analogy is to robot architecture; robots receive information through sensors and that data reflects both the status of the robot and the status of the world beyond the robot. Algorithms must integrate multiple data streams in order to both represent the world as accurately as possible and to control the robot's actuators as effectively as possible.

When multiple sensory modalities provide congruent data, the brain is more likely to “believe” the information to be true. Or, when asynchronous or ambiguous information is presented, the brain might reject the afferent information from one or more sensors as erroneous.

A common problem in navigational VR setups, simulator sickness, has its roots in discordant multisensory integration (Akiduki et al., 2003). When simulator sickness occurs, visual input might indicate movement, while the vestibular system does not. This mismatch in cross-modal sensory inputs generates a “Schrödinger cat situation” in the brain: the brain infers that the body is both static and moving. A clash of this kind must be resolved.

To tackle ambiguity in sensory information, the brain might seek higher probabilistic confidence in one interpretive state over the others; in this case between the person's location being in motion or stationary. For example, when subjects are seated, there is an increased number of skin pressure and proprioceptive sensors that add evidence that body position is static. The scales are tipped toward a fixed position interpretive state, so being seated can help reduce simulator sickness (Stoffregen and Smart, 1998). However, it also reduces the illusion of movement.

Similarly, visual experience can be modified, though that approach is usually less minimalist. For instance, VR headsets can be modified to optimize peripheral visual content in order to reduce simulator sickness (Xiao and Benko, 2016). Approaches to reduce simulator sickness can be invasive. One example is to stimulate the vestibular system directly with galvanic instrumentation (Lenggenhager et al., 2008).

In all these experiments, significant variation in individual responses has been observed. Cross-modal environmental and body interpretation varies from person to person.

Brains in subjects with extensive training in tasks that emphasize one modality will allocate more resources to that modality as a result of brain plasticity (Cotman and Berchtold, 2002). For instance, ballet dancers develop remarkable proprioceptive abilities, by which they are able to know very precisely where each limb of their body is, even with their eyes closed.

Internally, the sensory modalities are not exclusively pitted against one another. The brain also contains multisensory neurons that attend multiple inputs (Stein and Stanford, 2008). An audio-visual multisensory neuron, for example, will be more likely to fire when both excitatory stimuli are present and synchronous.

The multisensory system can enhance or depress the role of each unimodal stimuli exerting influence in a specific situation (Stein and Stanford, 2008). Typically, if one modality triggers more multisensory neurons than another, that modality is more likely to display dominance. In addition to cases of sensory modality dominance or suppression, cross-modal dynamics can help to explain synesthetic phenomena (Posner et al., 1976).

As noted earlier, visual-dominance is often associated with human cognition. This might be because, in addition to numerous unimodal visual neurons, many multimodal neurons are also influenced by visual stimulation: audio-visual, visuo-tactile, visuo-proprioceptive, visuo-vestibular (Bavelier and Neville, 2002; Shams and Kim, 2010).

Visual dominance enables bottom-up multisensory integration mechanisms that can be manipulated to generate body illusions leveraging visual stimulation. This is not only the basis of operation of many of the VR bodily illusions we have described (Normand et al., 2011; Piryankova et al., 2014), but it has also been shown to alter body perception in experiments that don't require VR. For instance, in the famous rubber hand illusion, participants believe that their hand has been replaced by a rubber hand through visuo-tactile synchronous stimulations (Botvinick and Cohen, 1998).

## Beyond bottom-up processing and a candidate for a theory of illusion in VR

Multisensory integration alone cannot explain why VR illusions are so strong. It only relies on the input of the afferent sensors at a specific moment and does not consider the history of previous states, while interactions with the real and virtual worlds are continuous. More complex prediction mechanisms take place in our brain.

**Figure 1:**
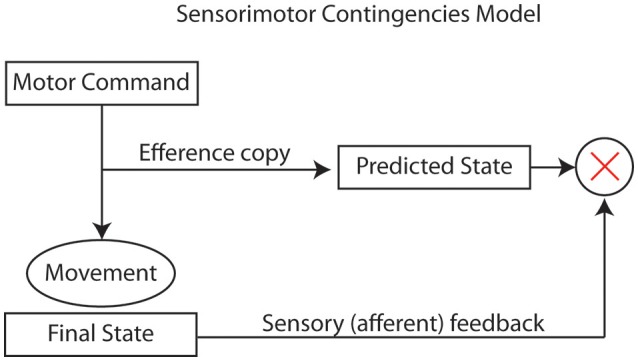
Sensorimotor contingencies model that enables VR illusion mechanisms.

Sensorimotor frameworks can be useful as explanations for VR's effective illusions. These frameworks rely strongly on the comparison of internal representations of the actual, desired, and predicted states of the external world after a motor action has been executed (Gallagher, 2000). If the afferent sensory input (with multisensory integration) matches the predicted state, then the brain is more likely to infer that the afferent input is correct. A simple model (Figure 1) can describe the functioning of sensorimotor contingencies that enable VR illusions.

A model of this type can also be used to describe the foundations of motor learning and the self-awareness of voluntary actions. This approach not only accounts for more passive VR illusions (such as in 1840 stereoscope or in modern 360 video), but also explains why these illusions are reinforced through intentional interaction and exploration of a virtual environment, and are even stronger when participants are embodied in an avatar.

When users move their head or limbs, through active, voluntary motor execution, and the predicted state in their brain matches the information that arrives through the sensory afferent modalities (e.g., vision, proprioception, audio…), then there is a strong VR illusion. The strength of the illusion ultimately derives from the powerful agency implications related to volition: “I am the initiator or source of the action” (Haggard et al., 2002).

This type of self-awareness model based on predictions can explain strong top-down manipulations of afferent feedback. An example is found in experiments with action binding mechanisms, where actions (such as pressing a button) and feedback (such as a delayed audio beep) can be perceived closer in time (Haggard et al., 2002). In these experiments, discordant afferent inputs are apparently recalibrated or suppressed in the brain in order to confirm a predicted state of the world (Haggard and Chambon, 2002): “I have a prediction, ergo this is my final state.” The illusion illustrated in such experiments is related to the illusions created in VR. The brain can “decide” that there is an error in measurement in order to reinforce a preference for a predicted outcome.

These top-down agency mechanisms that have been shown to increase tolerance to latencies in certain settings (up to 200 ms; Haggard and Chambon, 2002), have implications to VR experiences. Proprioceptive experiences can be manipulated in this way when reaching for objects in VR (Azmandian et al., 2016). Producing self-attribution of retargeted motions strong enough to ignore associated proprioceptive drifts if the tactile feedback is coherent with the visual input (Kokkinara et al., 2015; Azmandian et al., 2016).

When does a top-down mechanism fail? The brain will reject an illusion when the discordance between afferent sensory inputs and the predicted/intended state become too extreme. This failure mode of VR can be described as a semantic violation (Padrao et al., 2016). The degree of failure can be measured as an increase in perceived latency between intention and a perceived action (Haggard et al., 2002).

In sum, sufficient results exist to describe the broad underlying mechanisms that enable VR experiences to be internalized as real. Continuous bottom-up multisensory integration is modulated by complex cognitive predictions (Slater, 2009; Blanke, 2012). Predictions can be reinforced through interactions so that the brain might even “correct” some sensory deficiencies in order to match its predicted states using top-down manipulations (Haggard et al., 2002). These corrections are so powerful that can alter the sense of agency and produce self-attribution of avatar actions into participants (Banakou and Slater, 2014).

## Partial awareness of illusion

This model does not address varying levels of partial awareness that users report and demonstrate during their exposure to VR. Even though participants are aware at all times that they are in a computer simulation, evidence suggests that being exposed to certain scenarios—particularly when one's own sense of self is manipulated through altered avatar design—can produce non-conscious effects; these might be perceptual or behavioral (Yee and Bailenson, 2007).

We tentatively assemble several mechanisms related to levels of partial awareness in VR illusions.

One approach to understanding partial awareness of illusion in VR concerns the human capacity to enable automatic cognitive mechanisms. Once a task is well-trained, the brain becomes less consciously aware of performing that task, so it is able to focus on other mental activities. People can walk and talk on the phone at the same time, for instance, though the ability of an individual to accurately assess their own capacity for multitasking is imperfect. We might think of the general VR illusion as being similar to walking in the above example. The modifications cognitive processes have taken on in order for the simulation to feel real have become unconscious background activities, as described by well-established theories (Haggard et al., 2002). Empirical examples of automatic mechanisms in VR have been found using EEG recordings, when participants activate their motor cortex as a response to a threat (González-Franco et al., 2014). But also through behavioral responses when participants interact as bystanders in a violent scenario (Slater et al., 2013) or in the presence of a moral dilemma (Pan and Slater, 2011).

Reducing semantic violations is an essential task in VR, but the softer quality of plausibility further strengthens illusions in VR (Slater, 2009). We can extrapolate that the more plausible an illusion is, the more likely it is to be processed unconsciously.

Based on the model, we hypothesize that cognitive and sensory saturation will change the level of awareness of some illusions, i.e., a sufficient quantity of “tricks” in VR, as described in the experiments referenced in this paper, might be compounded in order to overwhelm the ability of an individual to consciously keep track of some illusory aspects of an experience. Therefore, more illusions would be undetected and accepted as real than if they had been presented one at a time. This might happen particularly when performing a task requiring higher cognitive functions, in which the brain is so saturated that it has no more load to dedicate to the evaluation of basic perceptual information. Further experiments would be needed to validate this hypothesis.

We are not yet proposing a model to explain in a comprehensive way how brain tolerance, automatic processing, and saturation might trigger different levels of awareness of a VR illusion. Incorporating further awareness mechanisms to our current model would probably require a more complex approach including more recent ideas from machine learning.

However, our model, based on classical, established theories, is useful for describing how VR illusions come about in the first place.

## Discussion

In this paper, we first reviewed illusions that can take place in VR and then presented a neuroscientific model able to describe why and how they take place. We suggest that VR illusions occur when media instrumentation stimulates neural bottom-up multisensory processing, sensorimotor self-awareness frameworks, and cognitive top-down prediction manipulations and furthermore allows these to reconcile in such a way that semantic violations are infrequent.

This model of illusion in VR summarizes how VR research has interacted with established human neuroscience theories, while also suggesting and requiring new ideas. For instance, VR enables unprecedented experiments that are both broadly multisensory, and yet with few uncontrolled variables, in order to investigate whole-body cognitive mechanisms (Kilteni et al., 2015). Indeed, the model accommodates a wide range of ergo-centered research in VR, including not only multisensorial illusions but also potentially illusory/false memories (Osimo et al., 2015), such as memories of agency (Guadagno et al., 2007), conversations with avatars (Pan et al., 2012), and engaging in plot interventions (Yee et al., 2009).

In all these cases VR presents an expanded experimental platform that can be interpreted using a model composed of previously-established theories—and yet, VR also presents new experimental design constraints, such as the avoidance of disabling, unintended semantic violations. Experiments taking place in physical reality avoid that problem, since physical reality is presumed to be well-ordered, complete, and consistent.

We discussed the question of partial awareness of VR illusions and some potentially relevant cognitive mechanisms, but we concluded that it is still premature to incorporate these elements into the model.

VR has recently become widely available, and it is ever more urgent for varied stakeholders to understand what illusions can be created in VR; those with ethical, legal, or compassionate concerns will benefit from a compact framework for understanding these illusions.

For instance, one worrying scenario is that in the future, if one is completing a work assignment within a virtual world, experiencing a degree of cognitive saturation, one's avatar might also be slightly altered in relation to incidental portrayals of a product or a political candidate, in order to achieve a change in behavior that would benefit a third party without the user's knowledge. While variants of this type of effect have been observed in prior media, the cited experiments show that manipulative illusions could be remarkably powerful in VR. Examples of this implicit behavioral avatar manipulations include the increase in saving behaviors after being embodied in an older avatar, or the altered negotiation skills after being exposed to taller or shorter avatars (Yee et al., 2009).

The model suggests how the manipulative aspects of the VR illusion can be selectively weakened. It can also help to identify manipulation abatement strategies that are unlikely to work.

We hope that our model can be leveraged as a base to design future VR experiences. We expect that both scientists and creators will find it useful for understanding the implications of the VR scenarios that they design and the types of illusions they generate.

## Author contributions

All authors listed have made substantial, direct and intellectual contributions to the work, and approved it for publication.

### Conflict of interest statement

The authors declare that the current manuscript presents a balanced and unbiased review on the field of Virtual Reality. The authors however report their affiliation to Microsoft, an entity with a financial interest in the subject matter or materials discussed in this manuscript. The authors have conducted the review following scientific research standards.
